# Evaluation and Prediction on the Effect of Ionic Properties of Solvent Extraction Performance of Oily Sludge Using Machine Learning

**DOI:** 10.3390/molecules26247551

**Published:** 2021-12-13

**Authors:** Changchao Hu, Shuhan Fu, Lingfu Zhu, Wei Dang, Tingting Zhang

**Affiliations:** 1 Petroleum Exploration and Production Research Institute, SINOPEC, Beijing 100083, China; hucc2049@163.com; 2 Department of Environmental Science and Engineering, College of Chemical Engineering, Beijing University of Chemical Technology, Beijing 100029, China; 2018010383@mail.buct.edu.cn (S.F.); 18994065595@163.com (L.Z.)

**Keywords:** ionic liquid, deep eutectic solvent, oily sludge, extraction, machine learning

## Abstract

Oily sludge produced in the process of petroleum exploitation and utilization is a kind of hazardous waste that needs to be urgently dealt with in the petrochemical industry. The oil content of oily sludge is generally between 15–50% and has a great potential for oil resource utilization. However, its composition is complex, in which asphaltene is of high viscosity and difficult to separate. In this study, The oily sludge was extracted with toluene as solvent, supplemented by three kinds of ionic liquids (1-ethyl-3-methylimidazole tetrafluoroborate ([EMIM] [BF_4_]), 1-ethyl-3-methylimidazole trifluoro-acetate ([EMIM] [TA]), 1-ethyl-3-methylimidazole Dicyandiamide ([EMIM] [N(CN)_2_])) and three kinds of deep eutectic solutions (choline chloride/urea (ChCl/U), choline chloride / ethylene glycol (ChCl/EG), and choline chloride/malonic acid (ChCl/MA)). This experiment investigates the effect of physicochemical properties of the solvents on oil recovery and three machine learning methods (ridge regression, multilayer perceptron, and support vector regression) are used to predict the association between them. Depending on the linear correlation of variables, it is found that the conductivity of ionic liquid is the key characteristic affecting the extraction treatment in this system.

## 1. Introduction

A large amount of oily sludge has been produced in the process of crude oil exploration, production, transportation, storage and refining [1]. Oily sludge has become a serious environmental problem in many countries. It is reported that China produces about 3 million tons of oily sludge every year [2]. Due to the large amount of toxic substances such as petroleum hydrocarbons in oily sludge, it is listed as hazardous waste in many countries. Therefore, the treatment of oily sludge has become an urgent issue. Among the existing oily sludge treatment methods, solvent extraction method has a good application prospect with the advantages of simple operation, high efficiency and short treatment time [2]. However, a large amount of organic solvent needs to be consumed in the extraction process. Therefore, how to reduce the solvent loss in the extraction process and reduce secondary pollution is the key problem to be solved in the field of oil sludge extraction [3].

Ionic liquids (ILs) have been used to improve the solvent extraction of petroleum in oil sands and sludge because of their unique properties, non-volatile, high thermal stability, low price, high polarity, relatively high conductivity and easy operation in liquid state [4]. Painter et al. investigated the enhanced effect of three different ionic liquids [Bmmim][BF_4_], [Bmmim][CF_3_SO_3_] and [Bmim][CF_3_SO_3_] in solvent extraction for asphalt recovery from bituminous sands, and application of [Bmmim][BF] resulted in a yield of more than 90% [5]. In addition, the asphalt extraction rate of about 94% can be achieved by using the third generation ILs (deep eutectic solvents) to assist naphtha to extract asphalt from oily sand. Painter et al. also inferred that the increase of pH was conducive to the recovery of asphalt [6]. Further studies showed that [Emim][BF_4_] could improve the oil recovery rate from oil sands via solvent extraction by about 10%, while the surface tension of [Emim][BF_4_] was determined to be the key factor affecting the separation efficiency, followed by the viscosity [7]. In addition, [Emim][BF_4_] was successfully used as an auxiliary solvent extraction to recover oil from oily sludge and improved the oil recovery by about 9.4% [4]. Previous studies have reported that pH, surface tension, viscosity and charge in ionic liquids can significantly change the interaction between asphalt and sand [6,7]. However, the quantitative effect of solvent physical parameters on solvent extraction has not been systematically studied.

In recent years, with the continuous development of computer technology, some new techniques (such as artificial neural networks, genetic algorithms, evolutionary algorithms, etc.) have been applied to many fields for building predictive models, estimating the required parameters, evaluating the impact of parameters [8]. Yilmaz et al. has applied three models (multiple regression, artificial neural network and adaptive neuro fuzzy system) to predict the strength and elastic modulus of gypsum through physical parameters such as porosity and water content [9]. In addition, several studies have reported several methods for water flow prediction based on artificial intelligence models [10]. Maier et al. summarized the current situation and future direction of neural network in water resources prediction [11]. However, the application of machine learning method in oily sludge treatment has not been reported.

In this study, the effect of three different ionic liquids and three different deep eutectic solvents on the performance of solvent extraction of oily sludge was investigated. The relationship between the properties of the extraction solvents and the extraction efficiency of oily sludge was explored for the first time and the correlation equations were obtained by multiple regression, support vector regression (SVR) and multilayer perceptron (MLP) models. This research will provide a scientific basis for further exploring the application of extraction technology in oily sludge treatment.

## 2. Results and Discussion

### 2.1. Ionic Liquids and Deep Eutectic Solvents Physicochemical Properties

**Figure 1:**
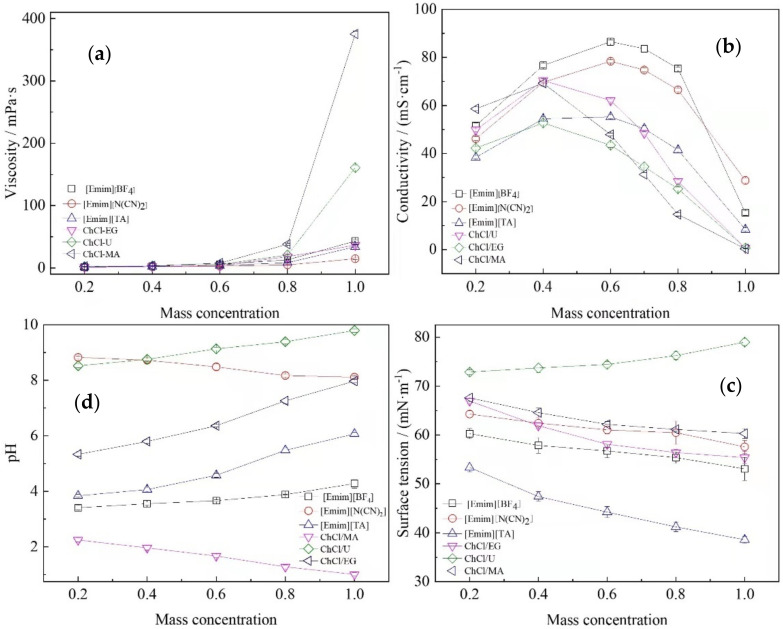
Physicochemical properties of the solvents with different concentrations: (**a**) viscosity; (**b**) conductivity; (**c**) surface tension; (**d**) pH.

Figure 1 showed the changes of some physicochemical parameters of ionic liquids and deep eutectic solvents with different mass concentrations. It can be seen from Figure 1a that after adding a small amount of water, the viscosity of the three ionic liquids and three deep eutectic solvents decreased significantly. When 20% water was added, the viscosity of ChCl-MA ionic liquid decreased by 90%, followed by the decrease of ChCl/U by 85%, and the viscosity change of ChCl-EG decreased by 50% at least. This is consistent with previous results on ionic liquid viscosity [12]. Then, the viscosity of the aqueous solution of ionic liquids decreased with the decrease of the mass concentration of ionic liquid and it finally tended to be close to the viscosity of water (1 mPa s) as shown in Figure 1a. This may be due to the fact that the addition of water reduces the interparticle resistance and the thickness of the boundary layer during the flow, making the flow more uniform and the gradual viscosity decrease. Unlike ionic liquids, the molecules in deep eutectic solvents are mainly connected by hydrogen bonds, forming a large number of hydrogen bonding network structures, and the addition of water is difficult to destroy the hydrogen bonding network structure, so the viscosity decreases more slowly [13]. After adding water, water molecules participate in the formation of hydrogen bonds, destroy the hydrogen bond grid structure and change the intermolecular van der Waals force, resulting in a significant decrease in the viscosity of the system [13,14].

Different from the change of viscosity, the conductivity of the extraction solvent solution (Figure 1b) increased greatly after adding a small amount of water. When 20% water was added, the conductivity of ChCl/U solution increased the most which became 45 times of the original; the least increase was for [Emim][N(CN)_2_], which also became 2.3 times of the original. The conductivity reaches a maximum at a mass concentration of 40–60% of the solvent. Then, the conductivity decreases with decreasing solvent concentration. As shown in Figure 1b, there were two obvious different regions in the ionic liquid aqueous: water rich region and salt rich region [14]. Mixtures of ionic liquids or deep eutectic solvent and water show the classical properties of concentrated salt solutions (i.e., the presence of a maximum conductivity). The conductivity increased sharply in the water rich region and decreased linearly in the salt rich region. It has been proved by Li et al. that solvents with higher dielectric constant tended to have a greater impact on the viscosity and conductivity of the solution [15]. It can be concluded that organic solvents would enhance the ion association of ionic liquids, while water significantly promoted the dissociation, which is consistent with previous research results [16].

The surface tension of ionic liquid and deep eutectic solvent varied approximately linearly with the increase of mass concentration, as shown in Figure 1c. With the decreasing concentration of ionic liquid and deep eutectic solvent, the surface tension of ionic liquid and deep eutectic solvent solution was close to that of water. The presence of surface tension can clearly explain the strength of intermolecular forces between molecules in mixtures [17]. Liu et al. raised that ionic liquids mainly act as surfactant in aqueous solutions. With the increase of ionic liquid concentration, micelles in the solution are also growing [14]. Sung et al. has proved that ionic liquid of 1-alkyl-3-methylimidazolium salt can form self-assembled aggregates in 3D network in aqueous solution, which makes the ionic liquid have very high activity on the surface [18].

As can be seen from Figure 1d, the pH of the extraction solvent was approximately linear with the mass concentration of ionic liquid mass. In a previous study by Guo et al., after the addition of water, ionic liquids and deep eutectic solvents appeared to be conjugated, and the ions in the conjugated form did not have an effect on the activity of water molecules [19]. The ionic liquid in inert form tended to form an ionic polymerization membrane, so that hydrogen ions cannot be dissociated into the solution. When water was gradually added, the liquid polymerization membrane slowly released hydrogen ions, resulting in a gradual change in pH [20].

### 2.2. Effect of Dosage of Ionic Liquid on Oily Sludge Extraction Efficiency

**Figure 2:**
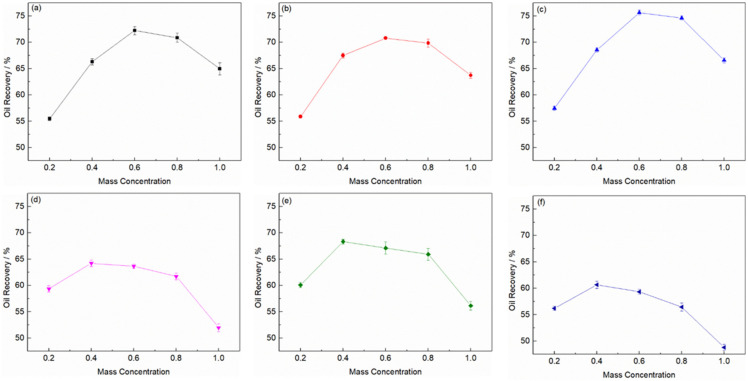
Oil recovery rate assisted with ionic liquid (**a**) [Emim][BF_4_]; (**b**) [Emim][TA]; (**c**)[Emim][N(CN)_2_]; and deep eutectic solvent (**d**) ChCl/U; (**e**) ChCl/EG; (**f**) ChCl/MA extraction.

The oil recovery rate assisted with various ionic liquids solvent extraction is shown in Figure 2. With the ionic liquid mass concentration increase in the extraction solvent, the oil recovery rate increased. For deep eutectic solvent ChCl/U, ChCl/EG and ChCl/MA, the oil recovery rate reached the highest with 40% of the liquid mass, among which ChCl/EG had the best performance. But for ionic liquid [EMIM][BF_4_], [EMIM][TA] and [EMIM][N(CN)_2_], it reached the maximum recovery rate when the mass concentration of individual ionic liquid was 60%. Among all ionic liquids, [Emim][N(CN)_2_] aqueous solution had the highest oil recovery ability (75.6%). It can also be seen that a small amount of water can significantly improve the ability of ionic liquid to extract oily sludge. As shown in Figure 2d, the extraction efficiency of ChCl/U can be increased by about 10% when 20% of water was added. However, when the mass concentration of water exceeded 60%, the content of extracted oil decreased. This may be because the addition of water changed the physical and chemical properties of ionic liquid solution. When the mass fraction of water was at 0–40%, the viscosity of ionic liquid decreased obviously, which was conducive to the rapid mixing of ionic liquid and oily sludge, as well as the entry of ionic liquid into the interface between soil and oil, so as to improve the extraction efficiency [13]. When the amount of water was further increased, the viscosity of ionic liquid did not change significantly [21]. However, the changes of conductivity, surface tension and pH may have a significant impact on the extraction process and further improve the extraction efficiency. Subsequently, with the continuous addition of water, the solvent was continuously diluted, resulting in the decline of extraction efficiency.

### 2.3. Machine Learning Analysis and Prediction Results

In order to accurately find the relationship between the physicochemical properties of ionic solution and extraction performance, viscosity, conductivity, pH and surface tension were selected as variables in this study for further prediction, as shown in Table 1.

**Figure 3:**
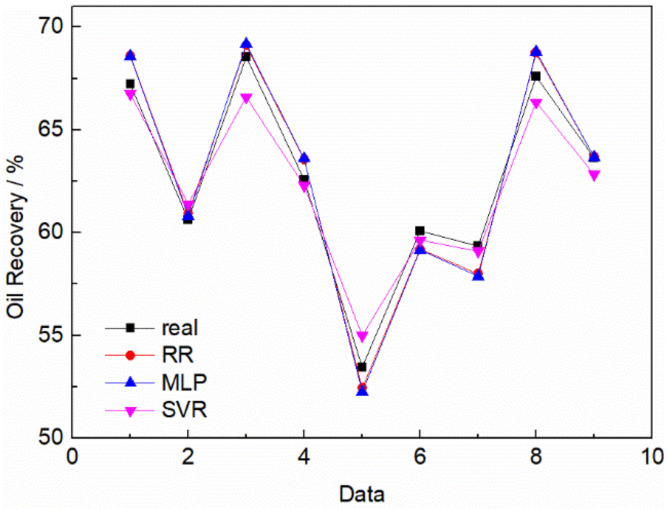
Comparison between prediction results of different machine learning algorithms and real experimental results. (‘real’ represents the experimental data, RR is the oil removal rate predicted by ridge regression algorithm, MLP is the oil removal rate predicted by multi-layer perceptron and SVR is the rate predicted by support vector regression algorithm method).

The supervised learning method was used to learn the data. All the data were divided into two groups: training set and test set. The ratio between them was 7:3 and the data of the training set was used to train the machine learning algorithm. After the training, the test set was used to test the training results. Then, the prediction results of machine learning are evaluated based on the fit of the experimental results to the computational results. Figure 3 showed the prediction of randomly selected experimental result samples after learning with three models respectively. The evaluation results of the model were shown in Table 2.

From the evaluation results, all three algorithms have high prediction accuracy. Among them, ridge regression algorithm has the best prediction accuracy, multi-layer perceptron algorithm and support vector regression algorithm have their own advantages and disadvantages. Nevertheless, as the study proceeds, the MLP and SVR algorithms may exhibit better predictive behavior if the amount of data and data complexity increases further. Ridge regression algorithm also gives the relationship between oil removal rate and various influencing factors:

$$f\left(\mathrm{Recovery}\right)=-0.0228\cdot f\left(\mu \right)-0.5989\cdot f\left(\gamma \right)+0.4735\cdot f\left(pH\right)+0.6568\cdot f\left(\sigma \right)+0.0615$$

where, f(·) represents the result after data center standardization.

The sign of ridge regression coefficient indicates that the physical and chemical properties had a positive or negative correlation with the final recovery, and the size of the coefficient indicates its ability to affect the recovery. Similar to the results of qualitative analysis, there was a roughly negative correlation between the viscosity and surface tension of the extraction solvents and the final oil recovery, that is, the smaller the viscosity and surface tension, the higher the oil recovery. The increase of the extraction solvent pH and conductivity will contribute to the recovery of oil. Li and Painter’s views were also consistent with this result [5,6,7]. The influence of physical properties on the extraction treatment of oily sludge was in the order of conductivity (*σ*) > surface tension (*γ*) > pH > viscosity (*μ*).

At the same time, it also shows that the mass fraction of water at 0–40%, the viscosity of the ionic liquid decreases significantly and the conductivity increases significantly, so the oil recovery rate is greatly improved. When the mass fraction of water added exceeds 40%, the viscosity of the ionic liquid solution basically remains unchanged, while the conductivity gradually decreases and the surface tension increases, leading to a decrease in the oil recovery rate. In practice, the concentration of extractant with the best extraction performance can be simulated according to the data of viscosity, conductivity, surface tension and pH of ionic solution at different concentrations.

## 3. Materials and Methods

### 3.1. Preparation of Ionic Liquids and Deep Eutectic Solvents

Chemicals including carbon tetrachloride, anhydrous sodium sulfate, cyclohexane and toluene were obtained from Sinopharm Chemical Reagent Co., Ltd., Beijing, China. Besides, the ionic liquids ([Emim][BF_4_], [Emim][TA] and [Emim][N(CN)_2_]) were provided by Lanzhou Institute of Chemical Physics, Chinese Academy of Sciences. The choline chloride (ChCl), urea (U), malonic acid (MA) and thylene glycol (EG) were purchased from Shanghai Aladdin Bio-Chem. Technology Co., Ltd., Shanghai, China. All chemicals were of reagent grade and used without further purification.

The new ionic liquids (low eutectic solvents) used in this study were prepared as described below which can be found elsewhere [22]. Choline chloride/urea (ChCl/U) low eutectic ionic liquid: Choline chloride and urea were dried to a constant weight in a vacuum drying oven. Then, they were mixed at a molar ratio of 1:2 and sealed. The liquid was heated in a water bath at 75 °C and stirred to obtain a homogeneous liquid. Choline chloride/ethylene glycol (ChCl/EG): Choline chloride and ethylene glycol were dried in a vacuum oven until a constant weight. Then, they were mixed and sealed at a molar ratio of 1:2, put into a water bath at 70 °C, and continuously heated and stirred until it became a homogeneous, colorless, and clear liquid. Choline chloride/malonic acid (ChCl/MA): Choline chloride and malonic acid were dried in a vacuum oven to constant weight, weighed at a molar ratio of 1:1 and then mixed and sealed. Then, the mixture was placed in a water bath and heated slowly to 80 °C, and kept at a constant temperature with continuous stirring until it was melted into a homogeneous, colorless and clear liquid. The above three deep eutectic solvents were stored in a vacuum oven at 70 °C.

### 3.2. Determination of Oil Content in the Oily Sludge

Oily sludge samples were taken from an oil field in northern China in September 2018. The content of oil, water and soil in oil sludge is measured by gravimetric method. The composition of the oily sludge is as follows: 34.55% water, 11.56% oil and 53.89% soil. First of all, 10.0 g of oily sludge was dried to constant weight at 105 ℃. The water content ($\omega_{w}$) is obtained from:

(1) $$\omega_{w}=\frac{m_{0}-m_{1}}{m_{0}}\times 100\%$$

where $m_{0}$ and $m_{1}$ were the mass of the raw oily sludge and dried oily sludge, respectively.

The dried oily sludge was mixed with the carbon tetrachloride (30 mL) followed by ultrasonication for 30 min and centrifugation for 5 min at 6000 rpm. The procedure was repeated several times until the carbon tetrachloride was colorless. The obtained sludge was dried to constant weight at 90 °C to remove the residual carbon tetrachloride. The oil content ($\omega_{o}$) is obtained from:

(2) $$\omega_{o}=\frac{m_{1}-m_{2}}{m_{0}}\times 100\%$$

where $m_{2}$ was the mass of the extracted oily sludge.

### 3.3. Extraction Procedures

All the separation systems were composed of oily sludge, IL and solvent. In this research, toluene was applied as the solvent. The oily sludge (5.0 g), ILs and toluene were mixed sequentially at a mass ratio of 1:2:1 in the 50 mL centrifugal tube, and stirred at 500 rpm for 15 min. Afterwards, the obtained mixture was centrifuged at 5000 rpm for 5 min to remove the upper solvent layer. The mixture was repeatedly washed with deionized water and centrifuged for 5 times to completely remove the extraction solvents remaining in the oily sludge. After that, it was dried in an oven at 105 °C to a constant weight [4,7]. All procedures were operated at 25 °C. The oil recovery was calculated from the mass loss of the oily sludge before and after the extraction. The recovery (θ) was calculated by:

(3) $$\theta =\frac{m_{0}^{\prime }-m_{1}^{\prime }}{m_{0}^{\prime }}\times 100\%$$

where the $m_{0}^{\prime }$ was the pre-treated oily sludge and the $m_{1}^{\prime }$ was the sludge after the treatment.

### 3.4. Physicochemical Analysis

The viscosity, conductivity and surface tension of three ionic solutions and three deep eutectic solvents were determined using a Brookfield DV-E viscometer with a thermostatic jacket, a Jenway 4071 conductivity meter with temperature and conductivity probes (probe unit constant = 1.01 cm^−1^) and a Kruss K11 tensiometer with a thermostatic jacket, respectively [23].

### 3.5. Data Processing and Analysis

#### 3.5.1. Ridge Regression

When the experimental results are affected by multiple factors, multiple regression can be employed to account for predicting the variance in an interval dependent [24,25]. The multiple regression can be used to learn more about the relationship between several independent or predictor variables and a dependent or criterion variable. The form of multiple regression is write as following:

(4) $$y=\beta_{0}+\beta_{1}x_{1}+\beta_{2}x_{2}+\cdots +\beta_{k}x_{k}$$

where $\beta_{1},\;\beta_{2}$, …, $\beta_{k}$ are the regression coefficients, $\beta_{0}$ is the constant. The equation can be written in the form of:

(5) y=Xβ 

where the *y*, *X*, β are in the form of matrix. To achieve the minimum variance, the loss function can be written as;

(6) $$J\left(\beta \right)=\sum {\left(y-X\beta \right)}^{2}\;$$

However, in this research, as the possibility of mutual influence between variables, the multiple regression has the problem of multicollinearity [26]. To solve the problem, the ridge regression includes a term consisting of positive penalty parameter 𝜆 times the model complexity into the loss function (Equation) [27].

(7) $$J\left(\beta \right)=\sum {\left(y-X\beta \right)}^{2}+\lambda {\left\|\beta \right\|}^{2}=\sum {\left(y-X\beta \right)}^{2}+\sum \lambda \beta^{2}\;$$

where the λ is the ridge regression coefficients. Meanwhile, the regression coefficients β is given by:

(8) $$\beta ={\left(X^{T}X+\lambda I\right)}^{-1}X^{T}y\;$$

where the *I* is the identity matrix.

In this research, the independent variables were viscosity (μ), pH and surface tension (γ). The data needs to be center standardized to remove the influence of different units on the coefficients.

#### 3.5.2. Support Vector Regression (SVR)

As one of the classification machine learning algorithms, support vector regression (SVR) is an algorithm based on statistical learning theory and the principle of structural risk minimization (SRM) [28]. The SVR has been widely applied for predicting complicated regression problems such as sludge and water treatment [29,30].

For the given training data $\left\{\left(x_{1},y_{1}\right),\;\left(x_{2},y_{2}\right)\;\ldots \;\left(x_{n},y_{n}\right)\right\}\subset X\times R$, where X represents the space of the input patterns (e.g., *X* = *R^n^*), the main aim in SVR is to find a hyperplane function f(*x*) that has the max ϵ deviation from the real obtained target values $y_{i}$ for all the training data [28]. Meanwhile, the function must be as flat as possible.

The errors less than ϵ are tolerated, while any deviation larger than ϵ is unacceptable. The function is taken in the form of:

(9) $$f\left(x\right)=\overset{n}{\underset{i=1}{\sum }}\omega_{i}\phi_{i}\left(x\right)+b\;\;\;$$

where the $\omega^{T}$ is the weights matrix, and b is the bias term. The ϕ represents a non-linear transformation of x from *R^n^* to higher dimensional space. The goal of SVR is to find proper ω and b to ensure the Euclidean norm ${\left\|\omega \right\|}^{2}$ has to be minimized with the function being as flat as possible [31]. The convex optimization problem of the minimized regression risk $R_{reg}$ is as following:

(10) $$\mathrm{minimize}\;R_{reg}=\frac{1}{2}{\left\|\omega \right\|}^{2}+C\overset{l}{\underset{i=1}{\sum }}\left(\xi_{i}+\xi_{i}^{*}\right)$$

(11) $$\mathrm{subject}\;\mathrm{to}:\;\left\{\begin{array}{c} y_{i}-\left(\omega^{T}\phi (x_{i})+b\right)<\epsilon +\xi_{i}\\ \left(\omega^{T}\phi (x_{i})+b\right)-y_{i}<\epsilon +\xi_{i}^{*}\\ \xi_{i},\;\xi_{i}^{*}\geq 0 \end{array}\right.$$

where the $\xi_{i},\;\xi_{i}^{*}$ are the slack variables to cope with otherwise infeasible constraints of the optimization problem. The constant *C* > 0 determines the both the flatness of the function and the tolerated deviations larger than ϵ.

By utilizing the Lagrange multiplier *α*, the optimization problem can be defined in Equation (12):

(12) $$L\left(\omega ,b,\alpha \right)=\frac{1}{2}{\left\|\omega \right\|}^{2}+\overset{n}{\underset{i=1}{\sum }}\alpha_{i}\left(y_{i}\left(\omega^{T}\phi_{i}\left(x_{i}\right)+b\right)-1\right)\;\;$$

By applying the Karush-Kuhn-Tucker (KKT) conditions to eliminate the product between constraints and dual variables at the optimal solution [29], the Equation (12) is transformed to:

(13) $$f\left(x\right)=\overset{n}{\underset{i=1}{\sum }}\left(\alpha_{i}-\alpha_{i}^{*}\right)\Phi \left(x_{i}\right)\cdot \Phi \left(x\right)+b\;$$

where, $\langle \Phi \left(x_{i}\right)\cdot \Phi \left(x\right)\rangle$ represents the inner product of the vector, which can be replaced by kernel equation, $k\left(x_{i},\;x\right)$ which can replace the dot product and calculate the dot product in the higher dimension feature space without knowing the form of the mapping function Φ [32].

(14) $$f\left(x\right)=\overset{n}{\underset{i=1}{\sum }}\left(\alpha_{i}-\alpha_{i}^{*}\right)k\left(x_{i},\;x\right)+b\;$$

There are different forms of the kernel function [29]. In this study, Gaussian kernel function is used with the SVR model. Where σ is the kernel scale, which regulates the effect of predictors variation on the kernel variation.

More details on the mathematics behind SVR models can be found in the literatures [33].

#### 3.5.3. Multilayer Perceptron (MLP)

**Figure 4:**
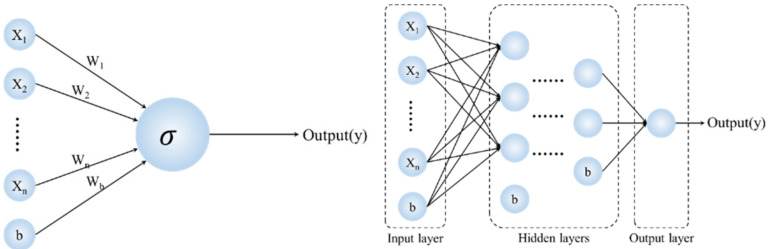
Schematic diagram of neurons (**left**) and multilayer perceptron (MLP) (**right**).

With the development of computer science and technology, artificial neural networks (ANN) are being used more and more widely [8]. Artificial neural networks are mathematical tools originally inspired by the way the human brain processes information. And a standard neural network (NN) is composed of many simple, connected processors called neurons (Figure 4). Each neuron produces a sequence of real-valued activations [34].

(15) $$y=\sigma \left(W^{T}X\right)\;\;$$

where the σ is the activation function, the matrix $W^{T}$ is the weight matrix. Through the activation function, the nonlinear factors can be introduced into neurons, so that the neural network can approach any nonlinear function arbitrarily. In this way, the neural network can be used in more nonlinear models.

Multi layer perceptron (MLP) can be obtained by combining multiple neurons, as shown in Figure 4. MLP is one of the most widely used neural network architectures for classification or regression problems. It can summarize complex or imprecise data. In this study, all data were standardized by the center and divided into two categories: training set (70%) and test set (30%). In this study, four layers of feedforward neural network architecture will be used, including one input layer (4 neurons), two hidden layers (6 and 2 neurons respectively) and one output layer (as shown in Figure 4). Layers are fully connected. The Multi Layer Perceptron (MLP) is one of the most widely used neural network architectures for classification or regression problems with complex or imprecise data [23,32]. The MLP networks are composed of multiple neurons and can be divided into an input layer, one or more hidden layers and an output layer. Each unit neuron is fully interconnected with weighted connections to neuron in the next layer.

In this research, all the experimental data was divided into two parts, the training set (70%) and the test set (30%). The MLP network transforms inputs to outputs through some nonlinear functions. The output of the network is determined by the activation function of the units in the output layer as follows [35].

(16) $$a_{i}^{l+1}=f\left[\sum \left(w_{ij}^{l}a_{j}^{l}\right)+b_{i}^{l}\right]\;$$

where the $w_{ij}^{l}$ is the interconnection parameter between the *j*th unit in the lth layer and the *i*th unit in the $\left(l+1\right)$th layer, i.e., the weight of the interconnection line; $b_{i}^{l}$ represents the bias term of the ith unit in the lth layer; $a_{i}^{l}$ is the output of the *i*th unit in the lth layer. When the  l=1, $a_{i}^{1}=x_{i}$, which is the *i*th input. And $f\left(\cdot \right)$ represents the activation function.

In this research, the tanh function is used as the activation function and is given as follows:

(17) $$f\left(z\right)=\tanh\left(z\right)=\frac{e^{z}-e^{-z}}{e^{z}+e^{-z}}$$

Based on the differences between the calculated output and the target value an error is defined as follows [35].

(18) $$\mathrm{LOSS}\left(y^{\prime },y,W\right)=\frac{1}{2}{\left\|y^{\prime }-y\right\|}^{2}+\frac{\alpha }{2}{\left\|W\right\|}^{2}$$

where the $y^{\prime }$ is the calculated data, *y* is the experimental data, *W* is the weight matrix, $\frac{\alpha }{2}{\left\|W\right\|}^{2}$ is the L^2^ regularization, and α is the regularization coefficient.

#### 3.5.4. Model Evaluation

The R^2^ was applied to evaluate the regression success of the model. Besides, the values account for (VAF) (Equation (18)) and root mean square error (RMSE) (Equation (19)) indices were calculated to control the performance of the prediction capacity of predictive model developed in the study [36].

(19) $$\mathrm{VAF}=\left[1-\frac{var\left(y-y^{\prime }\right)}{var\left(y\right)}\right]\times 100$$

(20) $$\mathrm{RMSE}=\sqrt{\frac{1}{N}\sum_{i=1}^{N}{\left(y-y^{\prime }\right)}^{2}}\;$$

where the y and $y^{\prime }$ are the measured and predicted values, respectively. The model will be perfect if the VAF is 100 and the RMSE is 0.

Also, the mean absolute percentage error (MAPE) was used to measure the accuracy in a fitted coefficients value in the model [25].

(21) $$\mathrm{MAPE}=\frac{1}{N}\sum_{i=1}^{N}\left|\frac{A_{i}-P_{i}}{A_{i}}\right|\times 100$$

where the $A_{i}$ and $P_{i}$ are the measured and predicted values, respectively.

## 4. Conclusions

In this study, the effects of three ionic liquids ([EMIM][BF_4_], [EMIM][TA], [EMIM][N(CN)_2_]) and three deep eutectic solvents (ChCl/U, ChCl/EG and ChCl/MA) on the treatment of oily sludge by solvent extraction at different concentrations were investigated. When 60% mass concentration [EMIM][N(CN)_2_] was used, the oil extraction efficiency was the best, and the oil recovery was 75.6%. In addition, it is also found that the addition of a moderate amount (0–40%) of water to the ionic liquid and deep eutectic solvent can improve the recovery of the extraction (12–32%). After normalizing the data between the four physicochemical property factors and the oil recovery rate, the machine learning results show that conductivity is the most important factor affecting the oil recovery efficiency, followed by pH, then surface tension, and viscosity has the least effect on the recovery efficiency. In this study, the viscosity of the ionic solution decreased significantly when the mass fraction of water added was between 0–40%, while the conductivity increased significantly, which would improve the recovery efficiency. The recovery is maximized when the conductivity is maximum and the viscosity is small. Ridge regression algorithm, multi-layer perceptron algorithm and support vector machine algorithm can effectively predict the effect of ionic liquid assisted extraction on oil sludge. The relationship between oil removal rate and various influencing factors is fitted, and the ridge regression algorithm is the closest to the experimental results. And with the further improvement and discovery of the database, the multi-layer perceptron algorithm and support vector machine algorithm still have a lot of room for improvement.

## Figures and Tables

**Table 1:** Different variable forms for predicting the performance of ionic solution extraction of oily sludge.

Variable	Extraction Method	Unit	Data Range
Viscosity (μ)	determined in Section 4 with the mass concentration of the extraction solvent 0.2 to 1.0	[mPa·s]	1–400
Conductivity (σ)		[mS·cm^−1^]	0–100
pH		-	1–10
Surface Tension (γ)		[mN·m^−1^]	30–80

**Table 2:** Evaluation results of different machine learning algorithms.

	VAF	RMSE	MAPE	R^2^
RR	95.72	0.95	1.37	0.96
MLP	95.14	1.01	1.44	0.95
SVR	95.50	1.03	1.38	0.95

## Data Availability

Not applicable.

## Sample Availability

1-ethyl-3-methylimidazole tetrafluoroborate ([EMIM] [BF_4_]), 1-ethyl-3-methylimidazole trifluo-ro-acetate ([EMIM] [TA]), 1-ethyl-3-methylimidazole Dicyandiamide ([EMIM] [N(CN)_2_]), choline chloride/urea (ChCl/U), choline chloride / ethylene glycol (ChCl/EG) and cho-line chloride/malonic acid (ChCl/MA) and oily sludge are available from the authors.
